# Associated factors of modern contraceptive use among women infected with human immunodeficiency virus in Enemay District, Northwest Ethiopia: a facility-based cross-sectional study

**DOI:** 10.1186/s12889-019-7675-3

**Published:** 2019-11-28

**Authors:** Yibeltal Bimrew Kebede, Tesfahun Taddege Geremew, Yohannes Mehretie, Ayenew Negesse Abejie, Liknaw Bewket, Endalkachew Dellie

**Affiliations:** 1 Dejen Primary Hospital, East Gojjam Zone Health Department, Amhara National Regional State Health Bureau, Bahir Dar, Ethiopia; 2 Department of Reproductive Health and Population Studies, School of Public health, College of Medicine and Health Sciences, Bahir Dar University, Bahir Dar, Ethiopia; 3 Ethiopian Field Epidemiology and Laboratory Training Program (EFELTP), Amhara Public Health Institute, Bahir Dar, Ethiopia; 4 Department of Reproductive Health, College of Health Sciences, Debre Markos University, Debre Markos, Ethiopia; 5 Department of Human Nutrition and Food Sciences, College of Health Sciences, Debre Markos University, Debre Markos, Ethiopia; 6 Center of Excellence for Human Nutrition, School of Human Nutrition, Food Science and Technology, Hawassa University, Hawassa, Ethiopia; 7 Department of Midwifery, College of Health Sciences, Debre Markos University, Debre Markos, Ethiopia; 8 Department of Health Systems and Policy, Institute of Public Health, College of Medicine and Health Sciences, University of Gondar, Gondar, Ethiopia

**Keywords:** Women, HIV infection, Contraceptive use, Ethiopia

## Abstract

**Background:**

The prevention of unplanned pregnancy among women infected with human immunodeficiency virus (HIV) is critical for the prevention of mother-to-child transmission (PMTCT) of HIV. Of the prevention strategies, deployment of modern contraceptives is principal one. However, there were limited facts on utilization of modern contraceptives and associated factors among HIV infected women, in particular of resource-limited settings in Ethiopia. Hence, we aimed to quantify the proportion of modern contraceptive utilization and the possible related factors among women infected HIV.

**Methods:**

A facility-based cross-sectional study was conducted on randomly selected 632 women infected with HIV from 05 February to 25 March 2018. Data on their treatment, socio-economic, and demographic background were collected through a structured interviewer administered questionnaire. Binary logistic regression model was fitted to identify the associated factors of modern contraceptive use among women infected with HIV.

**Result:**

We found 61.4% (95% CI, 57.6–65.2) were using modern contraceptives. Greater than four family size (AOR:2.17; 95%CI: 1.31–3.59), family planning counseling service (AOR: 2.37; 95% CI: 1.44–3.91), discussing contraceptive issues with sexual partner (AOR: 1.76; 95% CI: 1.12–2.77), history of giving birth (s) (AOR:2.21; 95%CI:1.20–4.05) and World Health Organization (WHO) clinical stage III or IV (AOR: 3.59; 95%CI: 1.37, 9.44) were positively associated with modern contraceptives use, whereas, older age (AOR: 0.45; 95% CI: 0.24–0.81) and being widowed (AOR: 0.34; 95% CI: 0.14–0.83), abridged the chances of modern contraceptives use.

**Conclusion:**

The prevalence of modern contraceptive use among women infected with HIV is low. Higher family size, counseling on contraceptives, discussing contraceptives issues with partner, history of giving births and WHO clinical stage III/IV were positively related with contraceptives use, whereas, older age and being widowed abridged the chances of contraceptives use among HIV infected women. Therefore, our findings support calls for the district health office and the antiretroviral treatment clinics of the resource-limited settings to work more on family planning counseling services and promoting more dialogues with sexual partners on modern contraceptives use.

## Background

Globally, an estimated 18.2 million women were infected with human immunodeficiency virus (HIV) in 2017 [1], and about 1.3 million women infected with HIV come to be pregnant every year [2]. World Health Organization (WHO) reported that an estimated 1.8 million children were living with HIV globally in 2017, whereas 180,000 children were newly infected with HIV [1]. Most of these children were living in sub-Saharan Africa and they were infected with HIV through mother-to-child transmission (MTCT) [3]. MTCT of HIV occurs when HIV is transmitted from a woman infected with HIV to her baby during pregnancy, labor or delivery, or through breastfeeding after giving of birth [4]. MTCT of HIV is a significant contributor to the burden of HIV, accounts for 9% of new infections globally [4]. HIV infection of infants results in early mortality or creates a lifelong chronic condition that greatly shortens life expectancy [4].

Many reproductive age women are simultaneously at risk for both HIV infection and unwanted pregnancy. This is more sever in sub-Saharan Africa, where the rate of unintended pregnancy among HIV infected women range from 51 to 84% [5, 6].

Hence, prevention of unintended pregnancies among HIV infected women is one of the main intervention strategies to prevent MTCT of HIV [7, 8]. Studies suggested that the effect of preventing unintended pregnancies among HIV infected women can be proportional or more pronounced than the contribution of antiretroviral treatment (ART) provision for pregnant women to prevent MTCT [9, 10]. Provision of appropriate counseling and contraceptive methods to WLHIV is recommended to ensure protection from unintended pregnancy [8, 11–13].

However, utilization of modern contraceptives among women infected with HIV is subjected with many different factors compared to HIV negative women of reproductive age. Evidences supported that freely open discussion about family planning with spouse and health workers [14–18], disclosure of HIV sero-status and discussion on fertility issue [19], being married, as well as older age of women [20], were substantial related factors of modern contraceptives use among HIV infected women. On the other hand, contradicting findings were also reported from Uganda and Kenya [18, 21] about the link between women’s educational status and modern contraceptives utilization.

Hence, special attentions are needed to reduce MTCT especially in low- and middle-income countries [1, 6, 22–24] by avoiding unintended pregnancies through the utilization of modern contraceptives [25]. Evidences indicated that contraceptive use averts 19.7% of MTCT, and 13.1% of deaths [10]. In the settings where HIV prevalence is high, management of sexual and reproductive health of women infected with HIV is critical to reduce HIV transmission and maternal mortality. In Ethiopia, an estimated 1.2% of all women and 1.5% of reproductive age women are living with HIV [26].

However, there is paucity of data on modern contraceptives use and its associated factors among HIV infected women in the childbearing age. Hence, this study aimed to determine the level of modern contraceptive use and its predictors among HIV infected women in the childbearing age.

## Methods

### Study design and setting

A facility-based cross-sectional study was conducted from 05 February to 25 March 2018 in Enemay District, Northwest Ethiopia. Enemay district is located 91 Kms due North from Debre Markos, the main town of East Gojjam Zone, and 245 km due East from Bahir Dar, the main town of Amhara national regional state. In 2018, the catchment population of the district was estimated to be about 200,000. The district had three ART centers (Bichena primary hospital, Yetmen health center and Bichena health center), 1878 reproductive age women infected with HIV were enrolled in the ART centers for HIV care services. The average daily patient visit of the ART centers was estimated to be 40 HIV infected women per day.

### Study population and eligibility

HIV infected women in the childbearing age who were receiving HIV care services from all the three ART centers of Enemay District were the study population. HIV infected women in the childbearing age who were at risk of pregnancy [i.e. women who were fecund, sexually active, and non-pregnant] were eligible for this study, whereas, pregnant women and sexually active HIV infected women in the childbearing age who were incapable to conceive for different reasons (for example, hysterectomy) were excluded from this study.

### Sample size determination and sampling procedure

The sample size of this study was determined using single population proportion formula:

$\mathrm{n}=\frac{Z^2\ast p\ast \left(1-p\right)}{(d)2}$ with the assumptions of 53.7% contraceptive users (p) among HIV positive women [17], 95% confidence level, and a maximum of 5% marginal error (d). Taking a non-response rate of 10% and 1.5 design effect, the final sample size was 632.

**Fig. 1:**
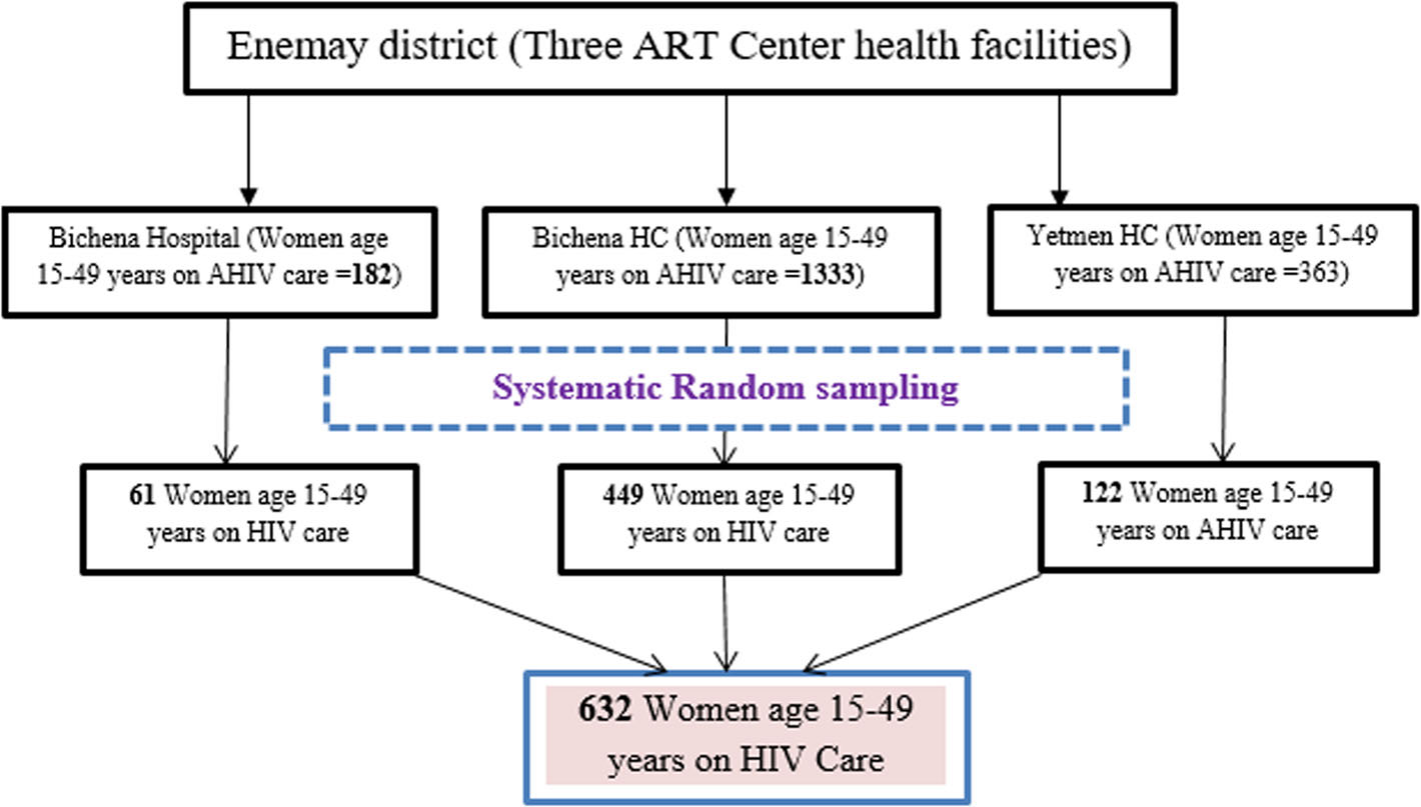
Schematic presentation of sampling procedure

The total sample size was allocated proportionally to the three ART centers in the District based on the number of HIV positive women in reproductive age who were on HIV care. Then, the study participants were selected through systematic random sampling of every other HIV infected women from the three study sites. The first HIV infected women coming to each ART center on the random start date was considered as a starting point of interviewing, and then we interviewed every other HIV infected women in the order of their visit until the calculated sample size in the respective ART center was saturated (Fig. 1).

### Data collection tools and procedures

Data were collected through interviewer-administered structured questionnaires, which were first prepared in English, then translated to the local language, Amharic, by language and public health experts. The data collection tool was pre-tested on 5% of the total sample (30HIV infected childbearing age women) out of the study area to ensure internal validity of the study. Then all the necessary corrections were also amended before the actual data collection based on the pre-test information we found. Three diploma nurses and two BSc nurses were employed as data collectors and supervisors respectively after training on the techniques of interviewing, handling ethical issues, maintaining confidentiality and privacy was provided by the principal investigator.

### Study variables and measurements

The dependent variable of this study was modern contraceptives use that was assessed through self-reported contraceptives use to delay or avoid pregnancy at the time of the study from a recognized family planning service provider facility. Modern contraceptive use was defined as the use of any method of modern contraceptive such as oral contraceptives/pills, injectable, Implants, intrauterine device (IUD), male or female condoms (restricted to those reporting “always” use or use frequently in all vaginal sexual relationships), and permanent methods such as vasectomy and Tubaligation [27, 28].

Independent variables included for this study were demographic and socio-economic characteristics (age, residence, religion, educational status, marital status, occupation, household’s monthly income, family size), reproductive health related characteristics (history of abortion, ever heard about modern contraceptives, type of modern contraceptives ever heard about, source of information, reasons to use modern contraceptive, reasons not to use modern contraceptive), and HIV care related characteristics (husband/partner sero status, disclosure of HIV, duration on ART, WHO clinical stage).

### Data management and statistical analysis

Data were checked for consistency, entered into Epi Info™ version 7.2, and then exported to SPSS version 25 for statistical analysis. Descriptive measures were computed to summarize the study participants and modern contraceptive utilization by background characteristics. Binary logistic regression model was fitted to identify factors associated with modern contraceptive use among HIV infected women. In the bi-variable analysis, independent variables with *p*-value less than 0.25 were included in multivariable logistic regression analysis to control possible confounders [29]. Model fitness was assessed with Hosmer and Lemeshow goodness of fit test, and Hosmer Lemeshow test greater or equal to 0.05 was considered to ensure goodness of fit of the model. Adjusted odd ratio (AOR) with 95% confidence level (CI) were used to declare statistically significant association between the outcome variable and independent variables.

### Ethical considerations

Ethical clearance and approval was obtained from the ethical review committee of Debre Markos University. Prior to interviewing, informed verbal consent was obtained from each participant after explaining the purpose and importance of the study. In case of minors (participants below 18 years old), verbal assent was obtained from the minors themselves and their parents or guardians. Participation was on a voluntary basis and the data were kept anonymous.

## Results

### Socio-demographic characteristics of the study participants

A total of 632 reproductive age women infected with HIV were involved in this study. The mean ± with its corresponding standard deviation of age of them was 31.1 ± 6.9 years and nearly half (45%) of the respondents were in the age group of 25 to 34 years. The samples were predominantly urban (60%) and orthodox Christian religion followers (74%). With regard to their occupation, 51% were farmers/Housewife. The result of this study also showed that 59% were married and 43% were cannot read and write of the total study participants (Table 1).

**Table 1:** Scio-demographic characteristics of study participants, Enemay District, Northwest Ethiopia, 2018 (*N* = 632)

Background characteristics	Frequency	Percent
Age group (years)		
15–24	123	19.5
25–34	286	45.2
35–49	223	35.3
Place of residence		
Urban	378	59.8
Rural	254	40.2
Religion		
Orthodox	465	73.6
Muslim	153	24.2
Protestant	14	2.2
Occupation		
Farmer/ housewife	325	51.4
Government employee	184	29.1
NGO/Private employee	45	7.1
Daily laborer	59	9.3
Students	19	3.0
Marital Status		
Single	93	14.7
Married	375	59.3
Divorced	102	16.2
Widowed	62	9.8
Educational Status		
Illiterate (can’t read and write)	273	43.2
Can read and write	129	20.4
Primary (grade 1–8)	87	13.8
Secondary (grade 9–12)	63	10.0
Certificate and above	80	12.6
Family size		
≤ 4	515	81.5
> 4	117	18.5
Household monthly income in Ethiopian Birr		
≤ 500	241	38.1
501–1500	187	29.6
1501–2500	76	12.0
2501+	128	20.3
Total	632	100.0

### Proportion of modern contraceptive use among HIV infected women

The result of this study showed that 388 (61.4% [95%CI: 57.6, 65.2]) participants were using modern contraceptive method, of which nearly 32% of contraceptive users were in the age group of 25–34 years. Nearly two-fifth (39%) of HIV infected women were married and urban residents (Table 2).

**Table 2:** Utilization of modern contraceptive by socio-demographic characteristics among women living with HIV in Enemy district, Northwest Ethiopia, 2018 (*N* = 632)

Socio-demographic characteristics	Total Participants		Modern Contraceptive Users	
	Frequency	Percent	Frequency	Percent
Age group (in years)				
15–24	123	19.5	80	12.7
25–34	286	45.2	200	31.6
35+	223	35.3	108	17.1
Residence				
Urban	378	59.8	246	38.9
Rural	254	40.2	142	22.5
Religion				
Orthodox	465	73.6	275	43.5
Muslim	153	24.2	102	16.1
Protestant	14	2.2	11	1.7
Occupation				
Farmer/ Housewife	325	51.4	200	31.6
Government employee	184	29.1	114	18.0
NGO/Private employee	45	7.1	28	4.4
Daily laborer	59	9.4	35	5.5
Students	19	3.0	11	1.7
Marital Status				
Single	93	14.7	62	9.8
Married	375	59.3	245	38.8
Divorced	102	16.2	56	8.9
Widowed	62	9.8	25	4.0
Education				
Illiterate (can’t read and write)	273	43.2	146	23.1
Can read and write	129	20.4	82	13.0
Primary (grade 1to 8)	87	13.8	63	10.0
Secondary (grade 9 to 12)	63	10.0	41	6.5
Certificate and Above	80	12.6	56	8.9
Family Size				
≤ 4	515	81.5	308	48.7
> 4	117	18.5	80	12.7
Income category				
≤ 500	241	38.1	127	20.1
501–1500	187	29.6	123	19.5
1501–2500	76	12.0	45	7.1
2501+	128	20.3	93	14.7
Total	632	100.0	388	61.4

Among HIV infected women who were using modern contraceptives, 49% of them had history of giving birth; of which, 7% had also experienced abortion. More than one-third of the participants (36%) discussed with their partner/husband about modern contraceptives, whereas almost all of the participants had ever heard about modern contraceptives (Table 3).

**Table 3:** Percentage of modern contraceptive users among women living with HIV by their reproductive health related characteristics, Enemay district, Northwest Ethiopia, 2018 (*N* = 632)

Reproductive health related characteristics	Total participants		Modern contraceptive users	
	Frequency	Percent	Frequency	Percent
History of giving birth				
No	134	21.2	78	12.3
Yes	498	78.8	310	49.1
Number of births (*n* = 498)				
1	105	21.1	52	8.2
2–3	237	47.6	162	25.8
4–5	118	23.7	76	12.0
6+	38	7.6	20	3.2
Number of children alive (*N* = 498)				
0	7	1.4	2	0.3
1–3	385	77.3	243	38.4
4–6	106	21.3	65	10.3
Ever experienced abortion				
No	541	85.6	342	54.1
Yes	91	14.4	46	7.3
Number of Abortions (*N* = 91)				
1	71	78.0	36	5.7
2+	20	22.0	10	1.6
Ever heard about Modern contraceptives				
No	0	0.0	0	0.0
Yes	632	100.0	388	61.4
Modern contraceptives methods ever heard about^a^				
OCP	499	79.0	326	51.6
Injectable	559	88.4	350	55.4
Implants	429	67.9	257	40.7
IUCD	169	26.7	91	14.4
Condoms	193	30.5	122	19.3
Source of information^a^				
Health Professionals Advice	580	91.8	367	58.1
Television	211	33.4	154	24.4
Radio	52	8.2	34	5.4
Neighbors	276	43.7	168	26.6
Reading books/leaflets	92	14.6	61	9.7
Counseled on modern contraceptives				
No	540	85.4	40	6.3
Yes	92	14.6	348	55.1
Ever discussed with husband/partner on contraceptives				
Yes	215	34.0	233	36.9
No	417	66.0	155	24.5
Reasons to choose currently used contraceptive method^a^				
Health professionals advice	170	26.9	170	26.9
Self interest	182	28.8	182	28.8
Partner agreement	14	2.2	14	2.2
Perceived less side effect	12	1.9	12	1.9
Easy to use	12	1.9	12	1.9
Total	632	100	388	61.4

^a^Since multiple answers are possible, the frequencies and percentages could not add up 632 and 100% respectively

**Fig. 2:**
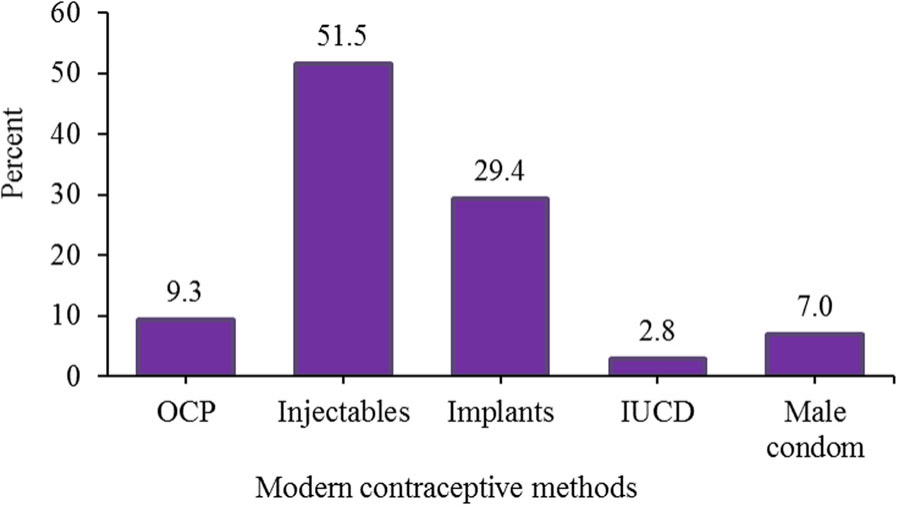
Percentage of women age 15–49 years living with HIV using modern contraceptive by method, Enemay district, Northwest Ethiopia, 2018 (*N* = 388)

Among the 388 modern contraceptive users, the most commonly used method was injectable (52%) followed by implants (29%) (Fig. 2).

### Reasons given for not using modern contraceptive methods

**Fig. 3:**
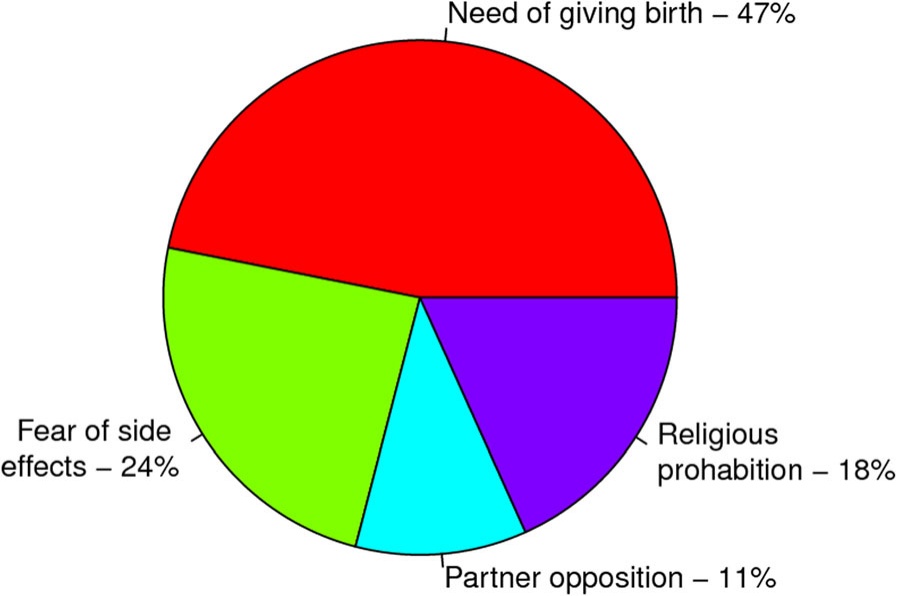
Percentage of WLHIV who were not using modern contraceptive by perceived reasons not to use, Enemay district, Northwest Ethiopia, 2018 (*N* = 244)

The need to give birth (47%) followed by fear of contraceptive method side effects (24%) were the frequently reported reports among HIV infected women who did not want to use modern contraceptives (Fig. 3).

The average length of time that women had been receiving care at the clinics was 4.6 years (±2.4SD). Forty three percent of modern contraceptive users had disclosed their sero-positivity status for HIV to close family members or communities, parents (37%) and 23% to partners/ husbands). Among the total of samples who were using modern contraceptives, 57% of them were in WHO clinical stage I/II (Table 4).

**Table 4:** Percentage distribution of modern contraceptive users by HIV related characteristics among women living with HIV in Enemy District, Northwest Ethiopia, 2018 (*N* = 632)

Background characteristics	Total Participants		Modern Contraceptive users	
	Frequency	Percent	Frequency	Percent
Disclosed of HIV status				
No	177	28.0	116	18.4
Yes	455	72.0	272	43.0
To whom it disclosed (*n* = 455)				
Partner	222	35.1	142	22.5
Parents	233	36.9	231	36.6
Others^a^	23	3.6	15	2.4
Husband/Partner Sero-status				
Reactive	263	41.6	254	40.2
Non-Reactive	29	4.6	20	3.2
Do not Know	340	53.8	114	18.0
Duration on ART (in years)				
≤ 2	160	25.3	99	15.7
> 2	472	74.7	289	45.7
WHO Clinical stage				
WHO stage I/II	598	94.6	360	57.0
WHO Stage III/IV	34	5.4	28	4.4
Total	632	100.0	388	61.4

^a^Community and close friends

### Factors associated with modern contraceptive use among HIV infected women

Table 5 summarizes the findings of our bivariate and multiple variable binary logistic regression analyses on the factors that associated with modern contraceptives use among HIV infected women. The middle age group were less likely to use modern contraceptives, women aged 35–49 years decreases the odds by 55% to use modern contraceptives compared with whose age was between 15 to 24 years (AOR = 0.45; 95% CI: 0.24–0.81).

**Table 5:** Factors associated with modern contraceptive utilization among women living with HIV in Enemay district, Northwest Ethiopia, 2018 (*N* = 632)

Background characteristics	Modern Contraceptive use		COR (95% CI)	AOR (95% CI)
	No, N (%)	Yes, N (%)		
Age group (in years)				
15–24	43 (6.8)	80 (12.7)	1.00	1.00
25–34	86 (13.6)	200 (31.6)	1.25 (0.80, 1.96)	1.17 (0.68, 2.00)
35–49	115 (18.2)	108 (17.1)	0.50 (0.32, 0.80)	0.45 (0.24, 0.81)*
Residence				
Urban	132 (20.9)	246 (38.9)	1.00	1.00
Rural	112 (17.7)	142 (22.5)	0.68 (0.49, 0.94)	0.75 (0.52, 1.08)
Marital Status				
Single	31 (4.9)	62 (9.8)	1.00	1.00
Married	130 (20.6)	245 (38.8)	0.94 (0.58, 1.52)	0.51 (0.25, 1.02)
Divorced	46 (7.3)	56 (8.9)	0.61 (0.34, 0.109)	0.54 (0.24, 1.18)
Widowed	37 (5.9)	25 (4.0)	0.34 (0.17, 0.66)	0.34 (0.14, 0.83)**
Family Size				
**≤** 4	207 (32.8)	308 (48.7)	1.00	1.00
> 4	37 (5.9)	80 (12.7)	1.45 (0.95, 2.23)	2.17 (1.31, 3.59)*
Ever had given birth				
No	57 (9.0)	78 (12.3)	1.00	1.00
Yes	187 (29.6)	310 (49.1)	1.21 (0.82, 1.78)	2.21 (1.20, 4.05)**
History of Abortion				
No	199 (31.5)	342 (54.1)	1.00	1.00
Yes	45 (7.1)	46 (7.3)	0.59 (0.38, 0.93)	0.67 (0.40, 1.12)
Ever counselled for modern contraceptives				
No	52 (8.2)	40 (6.3)	1.00	1.00
Yes	192 (30.2)	348 (55.1)	2.36 (1.51, 3.69)	2.37 (1.44, 3.91)*
Discussed contraceptive issues with partner				
No	184 (29.1)	233 (36.9)	1.00	1.00
Yes	60 (9.5)	155 (24.5)	2.04 (1.43, 2.91)	1.76 (1.12, 2.77)**
Disclosed of HIV status				
No	61 (9.7)	116 (18.4)	1.00	1.00
Yes	183 (29.0)	272 (43.0)	0.78 (0.54, 1.12)	0.73 (0.47, 1.13)
WHO clinical stages				
Stage I / II	238 (37.7)	360 (57.0)	1.00	1.00
Stage III / IV	6 (0.9)	28 (4.4)	1.76 (1.12, 2.75)	3.59 (1.37, 9.44)*

*P-Value <0.01, **P-Value <0.05

Marital status and parity as part of socio-demographic standard variables were also significantly affected the use of modern contraceptive use: Being widowed decreases the odds by 66% to use modern contraceptive as compared with singles (AOR: 0.34; 95% CI: 0.14–0.83), whereas, those who gave birth had the odds of 2.21 to use modern contraceptives than who don’t gave birth (AOR = 2.21; 95% CI: 1.20–4.05) Number of birth history had also significant relationship with modern contraceptives use; those women whose family size is four or more had the odds of 2.17 to use contraceptives than their counterparts (AOR = 2.17; 95%CI: 1.31–3.59).

Moreover, from the reproductive health related characteristics and counseling services, discussion with their partner were persistent predictors of modern contraceptives use. Holding the other variables constant, those women who got modern contraceptives had the odds of 2.37 to use modern contraceptives use than who don’t get counseling services (AOR = 2.37; 95% CI: 1.44–3.91). Whereas, women who had discussed with their partner about modern contraceptives had the odds of 1.76 to use modern contraceptives than their counterparts (AOR = 1.76; 95% CI: 1.12–2.77). Turning towards to their clinical characteristics we found WHO clinical stages were significant factors for modern contraceptives use; those women who developed WHO III/IV clinical Stages had the odds of 3.59 to use modern contraceptives than WHO I/II clinical Stages (AOR: 3.59; 95%CI: 1.37, 9.44) (Table 5).

We examined the data to see if our strongest associations were modified by residency or educational status. We found no evidence that the associations between modern contraceptive use, income, religion, occupation, other reproductive health and clinical characteristics.

## Discussion

This study set out to determine the proportion of modern contraceptive use among HIV infected women and to examine the associated factors during the course of their treatment. We found that the proportion of modern contraceptives use was 61.4%. Family size, FP counseling service, open discussion with partner on modern contraceptives issues, history of giving birth (s) and WHO Clinical stage II/IV, were all independently and positively associated with modern contraceptives use. Middle age (35–49 years) and being widowed reduced the odds of modern contraceptive use.

With regard to the proportion of modern contraceptives use, comparable findings were also reported from Southwest Ethiopia, 64% [15], and Western parts of Ethiopia at Gimbie town, 57% [30], and From Southern parts of Ethiopia at Yirgalem Hospital ART Centre, 62% [31].. However, higher than the current finding from Northwest parts of Ethiopia at Bahir Dar, 80% [32], and from Western Ethiopia at Nekemte town, 66.4% [33] and lower findings from Oromia region, Ethiopia, (46%) [34], Northern Ethiopia, 50% [35], 44% [36] and 46.3% [17], Northwest Ethiopia (48%) [37], Ghana, 43% [38], Nigeria,10% [39] and Kenya, 34% [40] was also reported. This variation among reports might be due to differences in background characteristics of the respondents, health service availability and accessibility, community awareness on modern contraceptives, presence of partners working on maternal health particularly on family planning services and health professionals’ health service provision experience.

The finding of this study was also higher than the study found on the general population of Ethiopia, 35% [41]. This can be possibly explained by that special attention for women on HIV care in terms of modern contraceptives service provision including contraceptive method supply, counseling services and others health care follow-ups. This may enhance modern contraceptives utilization among HIV infected women compared with the general population..

Despite, modern contraceptive methods were freely available in health facilities including at the ART clinics, nearly two-fifth of HIV infected women do not used any of the modern contraceptives methods. However, the Ethiopian HIV/AIDS prevention and control program national guidelines advocate for dual family planning methods to prevent HIV/STI transmission and unintended pregnancies for HIV infected individuals, the current study reported that only 4% of women used male condom as dual method of contraception. Parallel finding was also reported from previous survey conducted in the Ethiopian general population [41]. However a bit higher findings was also reported from Northern Ethiopia (59.9%) [17], from Northwest Ethiopia (8%) [35] and Southwest parts of Ethiopia (31.4%) [15].

In the current study, the finding revealed that women infected with HIV had different reasons not to use modern contraceptives. They explained that, the need of giving birth, fear of side effects, religious prohibition and partner opposition were the main reasons not to use modern contraceptives method. This finding was supported with a study done at the Northwest parts of Ethiopia in University of Gondar specialized teaching hospital [35].

Regarding the associated factors, many of the findings are unsurprising and in line with those recent studies related with the current study. For example, the issue of less modern contraceptives utilization among the middle age groups of HIV infected women was also reported from the northwest parts of Ethiopia at University of Gondar specialized Hospital [35], from the southwest parts of Ethiopia at Mizan-Tepi Teaching and Referral Hospital [15] and from the northern parts of Ethiopia at Tigray zonal hospitals [17]. This is possibly explained by that women under this age group may expect that there may be physiological cessation of menses and fear of the side effects while age increases. It can also be possibly justified by that misconception, cultural and religious barriers may affect the attitude of HIV infected women towards modern contraceptive utilization [42]. Similarly, widowed marital status was negatively associated with modern contraceptive utilization, which is also consistent with a recent study finding from the Southwest Ethiopia at Mizan-Tepi Teaching and Referral Hospital [15]. This is because of that the probability of doing sexual intercourse among widowed HIV infected women is negligible, which also indirectly increases the probability of not using modern contraceptives among them.

On the other hand, having a family size of four or more was statistically significant in relation to modern contraceptives use among women on HIV care, which was in line with a finding in Gimbie town, West Ethiopia [30]. This can be further explored that women who have higher number of family size might have lesser desire to have children than those who had four or less family size.

The strong association we found between discussion of contraceptive issues with their partner/husband and modern contraceptives use was also already established from northern Ethiopia [14, 17], Addis Ababa, Ethiopia [16] and Southwest Ethiopia [15], which might be because of these women’s freedom to negotiate their partners in decisions of birth limiting or spacing and safe sexual practice.

Moreover, getting family planning counseling services had increased odds of contraceptives utilization, which could also be explained by the assumption that women could get advice on the importance of modern contraceptives use, how to negotiate with sexual partner and risk reduction strategies.

Once more, this study showed that women who were at advanced stage of the disease had also increased odds of modern contraceptives utilization compared with their counterparts, which can also be explained by that due to fear of pregnancy related complications and the occurrence of opportunistic infection as clinical stage increases.

This study acknowledged some limitations. First, the study design makes it difficult to establish causality. This is because the cross sectional nature of this study in which cause-effect relationship couldn’t be established [43, 44]. Second, though there are wide ranges of factors, which affect utilization of contraceptive methods among HIV positive women, only individual level factors were addressed in this study. Hence, this is the interest of the authors to warrant further investigation to explore about the relationship between service providers related factors, structural barriers and modern contraceptive use among HIV infected women.

## Conclusion

Modern contraceptive use among women infected with HIV is better than the general population in Ethiopia. Higher family size, counseling on contraceptives, discussing contraceptive issues with partner, history of giving births and WHO clinical stage III/IV were positively associated with contraceptive use, whereas, older age and being widowed reduced the odds of contraceptive use among HIV infected women. Therefore, our findings support calls for the district health office and the antiretroviral treatment clinics of the resource-limited settings to work more on family planning counseling services and promoting discussions with sexual partners on modern contraceptives use.

## Data Availability

The datasets are available from the corresponding author on reasonable request.

## Abbreviations

AIDS: Acquired immune deficiency syndrome
AOR: Adjusted odd ratio
ART: Anti-retroviral treatment
CI: Confidence interval
COR: Crude odd ratio
FP: Family planning
HIV: Human immunodeficiency virus
IUCD: Intra-uterine contraceptive devices
OCP: Oral contraceptive pills
PMTCT: Prevention of mother-to-child transmission
SD: Standard deviation
WHO: World health organization
WLHIV: Women living with HIV
